# Effects of Sorghum [*Sorghum bicolor* (L.) Moench] Crude Extracts on Starch Digestibility, Estimated Glycemic Index (EGI), and Resistant Starch (RS) Contents of Porridges

**DOI:** 10.3390/molecules170911124

**Published:** 2012-09-17

**Authors:** Dilek Lemlioglu-Austin, Nancy D. Turner, Cassandra M. McDonough, Lloyd W. Rooney

**Affiliations:** 1 Novozymes NA, 77 Perrys Chapel Church Road, Franklinton, NC 27525, USA; 2 Department of Nutrition and Food Science, Texas A&M University, College Station, TX 77843, USA; Email: n-turner@tamu.edu; 3 Department of Soil and Crop Sciences, Texas A&M University, College Station, TX 77843, USA; Email: casstx@gmail.com (C.M.M.); lrooney@tamu.edu (L.W.R.)

**Keywords:** sorghum phenolics, starch digestibility, Estimated Glycemic Index, resistant starch

## Abstract

Bran extracts (70% aqueous acetone) of specialty sorghum varieties (tannin, black, and black with tannin) were used to investigate the effects of sorghum phenolic compounds on starch digestibility, Estimated Glycemic Index (EGI), and Resistant Starch (RS) of porridges made with normal corn starch, enzyme resistant high amylose corn starch, and ground whole sorghum flours. Porridges were cooked with bran extracts in a Rapid Visco-analyser (RVA). The cooking trials indicated that bran extracts of phenolic-rich sorghum varieties significantly reduced EGI, and increased RS contents of porridges. Thus, there could be potential health benefits associated with the incorporation of phenolic-rich sorghum bran extracts into foods to slow starch digestion and increase RS content.

## 1. Introduction

Obesity and diabetes are among the most important medical problems in the World [1]. Readily digestible carbohydrates lead to rapidly elevated blood glucose levels and insulin secretion, both of which contribute to the health complications caused by diabetes. Glycemic index (GI) ranks carbohydrate-containing foods on how quickly and how much they elevate blood sugar levels. Foods can be classified as having a low (<55), intermediate (55–70), or high GI (>70). GI can be estimated by *in vitro* rate and extent of starch digestibility, which is called Estimated Glycemic Index (EGI) [2,3].

Foods with a low GI and higher RS help slow absorption of carbohydrates and prevent extreme blood glucose fluctuations [4,5]. RS is the sum of any starch and starch degradation products not absorbed in the small intestine, because RS escapes digestion; it contributes to the fermentable carbohydrates entering the colon and provides a source of nutrients for colonic bacteria [6]. As these microorganisms metabolize the carbohydrate material via fermentation, the colonic pH is lowered and short chain fatty acids (SCFA) (e.g., acetate, propionate, and butyrate) are released. Because of these attributes, RS may also reduce the risk for colon cancer, obesity, diabetes and inflammatory bowel disease [1,7,8].

Le Bourvellec *et al.* [8], Davis and Hoseney [9] and Rahman and Richards [10] found that sorghum condensed tannins can bind starch and polysaccharides. Because phenolic compounds are known to complex with proteins and carbohydrates in foods resulting in structures that impact digestibility [9,10], it is possible to reduce starch digestibility and elevate RS content of flours by adding these natural inhibitors to food formulations. The interactions are mostly non-covalent, hydrophobic interactions, which are governed by the molecular weight, solubility, size, and conformational flexibility of phenolic compounds, proteins, and starch [11,12]. In addition, phenolic compounds can also directly bind to digestive enzymes (sucrase, amylases, trypsin, chymotrypsin and lipase), decreasing enzyme functionality [13,14,15], and further slowing the rate of protein and starch digestion.

Specialty sorghum varieties contain various types of phenolic molecules, including condensed tannins (polymers of flavan-3-ols) and anthocyanins (luteolinidin and apigeninidin) [16,17]. Davis and Hoseney [9] reported that isolated condensed tannins from tannin sorghum inhibited α-amylase, and bound to starch to varying degrees. Daiber [18] and Beta *et al.* [19] found that condensed tannins were responsible for reducing starch breakdown and sugar production during brewing due to inactivation of malt amylases. EGI of porridges and extruded products made with tannin-containing sorghums were more slowly digested *in vitro* than those made with non-tannin sorghum or with decorticated tannin-sorghums [20]. Jankowski *et al.* [21] reported daily consumption of grape anthocyanins decreased glucose concentrations in urine and blood of diabetic rats. Tsuda *et al.* [22] provided evidence that anthocyanins extracted from purple corn inhibited increases in both body weight and adipose tissue weight of rats consuming high-fat diets. Typical symptoms of hyperglycemia, hyperinsulinemia, and hyperleptinemia provoked by a high-fat diet did not occur when mice also ingested isolated anthocyanins. The researchers suggested that anthocyanins, as a functional food component, can aid in the prevention of obesity and diabetes. Hargrove *et al.* [23] compared the ability of simple flavonoids and proanthocyanidins in *Sorghum bicolor* bran (sumac sorghum bran) extracts to inhibit enzymes *in vitro*. In particular, inhibition of α-amylase can reduce the glycemic effect of dietary starches. Proanthocyanidin-rich sumac sorghum bran extract inhibited α-amylase at a lower concentration (50% inhibitory concentration [IC_50_] = 1.4 μg/mL) than did proanthocyanidin-free black sorghum bran extract (IC_50_ = 11.4 μg/mL).

Recently, we demonstrated that phenolic-rich specialty sorghum brans decreased starch digestibility of endosperm porridges, regardless of endosperm hardness, except for bran from black sorghum, which may have been due to its unique bran structure [24]. It would be possible to determine the specific effects of condensed tannins and anthocyanins on starch digestibility without the confounding factor of bran physical structure if extracts from the brans were used. Thus, this study was designed to investigate the effects of specialty sorghum bran extracts on starch digestibility, EGI, and RS of corn starch and enzyme resistant high amylose corn starch. The extracts were also added to their corresponding whole sorghum flours to determine if it was possible to suppress starch digestibility of foods prepared from these flours even further than might occur with the compounds present in the flours.

## 2. Results and Discussion

Brans of the specialty sorghum varieties are a more dense source of total phenols and condensed tannins than the whole sorghum grains [24]. Extraction recovered 69%–100% of the phenols and 92%–97% of the tannins, and 99% of the anthocyanins (Table 1) detectable in the sorghum brans.

**Table 1:** Total phenol and tannin contents of sorghum brans before and after extraction (residues).

Sorghum Brans	Total phenols (mg/g gallic acid eq.)	Tannins (mg catechin eq./g)	Anthocyanins (mg/g)
White corn	2.8 ± 0.1 ^f,1^	0.0 ± 0.0 ^d^	0.0 ± 0.0 ^c^
**White corn residue**	**0.1 ± 0.0 ^g^**	**0.0 ± 0.0 ^d^**	**0.0 ± 0.0 ^c^**
White bran	4.3 ± 0.7 ^e^	0.0 ± 0.0 ^d^	0.0 ± 0.0 ^c^
**White bran residue**	**0.0 ± 0.0 ^g^**	**0.0 ± 0.0 ^d^**	**0.0 ± 0.0 ^c^**
Black bran	15.0 ± 1.2 ^c^	2.0 ± 0.4 ^c^	4.2 ± 1.2 ^a^
**Black bran residue**	**4.6 ± 0.8 ^e^**	**0.0 ± 0.0 ^d^**	**0.0 ± 0.0 ^c^**
Black with tannin bran	24.0 ± 2.4 ^b^	24.0 ± 4.3 ^b^	2.0 ± 0.1 ^b^
**Black with tannin bran residue**	**4.6 ± 0.4 ^e^**	**1.8 ± 0.3 ^c^**	**0.0 ± 0.0 ^c^**
Tannin bran	40.3 ± 3.2 ^a^	75.0 ± 4.8 ^a^	0.0 ± 0.0 ^c^
**Tannin bran residue**	**7.2 ± 1.0 ^d^**	**2.6 ± 0.7 ^c^**	**0.0 ± 0.0 ^c^**

^1^ The data represent the means ± SD of three determinations. Values with the same letter in one column are not significantly different (*p* = 0.05) from each other. Values are reported on a dry basis.

### 2.1. The Effects of 70% Aqueous Acetone Extracts from Sorghum Bran on Starch Digestibility of Corn Starch Porridges

Corn starch was cooked with sorghum bran extracts to investigate extract effects on starch digestibility. Unlike the responses observed when white tannin bran extract was added to the corn starch porridges, porridges cooked with phenolic-rich bran extracts had significantly (*p* < 0.05) lower breakdown and setback in the Rapid Visco-analyser (RVA) test (Table 2), suggesting a possible reaction between extracts components and starch. Also, another work conducted in our lab by Dlamini [12] reported that processing by extrusion cooking and porridge-making significantly reduced measurable phenols, tannins and antioxidant activity of sorghum flours. Extrudates and porridges had lower amounts of tannins compared to the grain, and the most decrease was in tannin polymers, DP > 8. This was an indication that these polymers interacted with the food matrix, such as starch.

**Table 2:** Pasting properties of corn starch porridges cooked with sorghum bran extracts by Rapid Visco Analyzer.

Porridges	Peak Viscosity (RVU)	Trough (RVU)	Breakdown (RVU)	Final Viscosity (RVU)	Setback (RVU)	Peak time (min)	Pasting Temp. (°C)	Peak Temp. (°C)
Corn starch	451 ± 30 ^a,1^	154 ± 20 ^c^	297 ± 14 ^a^	468 ± 29 ^a^	314 ± 25 ^a^	4.6 ± 2.1 ^a^	73.9 ± 3.5 ^a^	94.8 ± 5.6 ^a^
Corn starch + white bran extract	426 ± 39 ^a^	136 ± 15 ^d^	290 ± 15 ^a^	405 ± 24 ^b^	269 ± 48 ^b^	4.7 ± 0.9 ^a^	75.2 ± 4.3 ^a^	94.5 ± 4.4 ^a^
Corn starch + tannin bran extract	401 ± 36 ^a^	210 ± 13 ^a^	191 ± 12 ^c^	353 ± 17 ^c^	142 ± 18 ^d^	4.9 ± 1.9 ^a^	74.2 ± 4.0 ^a^	93.3 ± 4.3 ^a^
Corn starch + black with tannin bran extract	412 ± 25 ^a^	175 ± 15 ^b^	237 ± 18 ^b^	365 ± 40 ^c^	159 ± 25 ^c^^d^	5.0 ± 2.0 ^a^	75.4 ± 1.9 ^a^	93.6 ± 7.2 ^a^
Corn starch + black bran extract	416 ± 26 ^a^	167 ± 14 ^b^	249 ± 33 ^b,l^	334 ± 21 ^c^	198 ± 48 ^c^	4.8 ± 1.2 ^a^	74.1 ± 2.1 ^a^	94.9 ± 4.6 ^a^

^1^ The data represent the means ± SD of three determinations. Values with the same letter in one column are not significantly different (*p* = 0.05) from each other.

**Figure 1:**
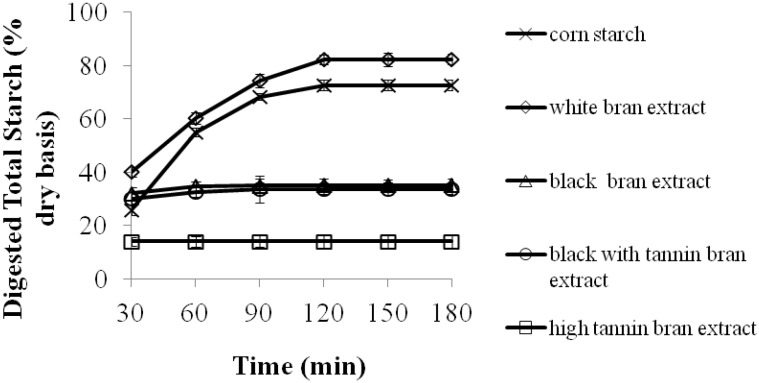
Starch digestibility of corn starch porridge made with sorghum bran extracts as a function of time (porridges used the equivalent of 50 mg of starch on a dry weight basis). Values are means ± standard error of *n* = 3 samples per treatment.

In general, phenolic-rich sorghum bran extracts significantly (*p* < 0.05) decreased overall corn starch digestion. Black bran or black with tannin bran extracts had similar effects on corn starch digestibility, while the extract from tannin bran dramatically reduced corn starch digestibility (Figure 1). However, white bran extract had significantly (*p* < 005) higher starch digestibility than corn starch porridge (Figure 1). Gularte and Rosell [25] reported a significant negative correlation between final viscosity and rapidly digested starch, while there is a significant positive correlation between final viscosity and slowly digestible potato starch. These differences were caused by easier enzyme penetration on softer gels than firmer gels. White bran extract porridges had a lower viscosity (final viscosity) values upon cooling to 50 °C in RVA (Table 2), which is an indication of a weaker gel formation, and may result in easier enzyme penetration.

Corn starch porridges cooked with extracts from the black and black with tannin bran had similar EGIs, while porridge made with the tannin bran extract had the lowest EGI (Table 3). Extracts from specialty sorghum brans significantly (*p* < 0.05) increased RS contents of corn starch porridges, compared to extracts from white sorghum bran (Table 3).

**Table 3:** Estimated Glycemic Index (EGI) and Resistant Starch (RS) Contents of Corn Starch Porridge Cooked with Sorghum Bran Extracts.

Porridges	Estimated Glycemic Index (EGI)	Resistant Starch (% dry weight basis)
Corn starch	90.0 ± 5.6 ^b,1^	0.0 ± 0.0 ^c^
Corn starch + white bran extract	98.0 ± 4.2 ^a^	0.2 ± 0.0 ^c^
Corn starch + high tannin bran extract	49.0 ± 2.4 ^d^	14.9 ± 0.9 ^a^
Corn starch + black bran extract	67.0 ± 3.6 ^c^	9.1 ± 0.6 ^b^
Corn starch + black with tannin bran extract	65.0 ± 6.3 ^c^	10.6 ± 1.0 ^b^

^1^ The data represent the means ± SD of three determinations. Values with the same letter in one column are not significantly different (*p* = 0.05) from each other. Values are reported on a dry basis.

### 2.2. The Effects of 70% Aqueous Acetone Extracts from Sorghum Brans on Digestibility of High Amylose Corn Starch Porridges

**Figure 2:**
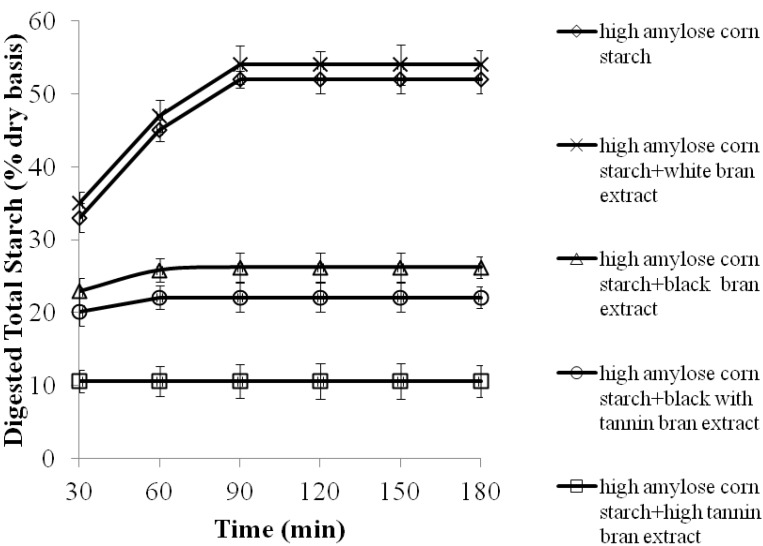
Starch digestibility of high amylose corn starch porridge made with sorghum bran extracts as a function of time (porridges used the equivalent of 50 mg of starch on a dry weight basis). Values are means ± standard error of *n* = 3 samples per treatment.

High amylose corn starches are used as a substitute for flour to add dietary fiber, lower GI, improve insulin sensitivity, and promote digestive health in foods. Forty-five to sixty percent of high amylose has been reported to be indigestible, and is being labeled as dietary fiber [6,7]. As expected, starch digestibility of the high amylose corn starch (enzyme resistant) was less than for normal corn starch [6,7,15]. The addition of extracts from the sorghum brans (except white bran) significantly (*p* < 0.05) decreased overall starch digestibility, EGIs and increased RS contents of high amylose corn starch porridges. The largest reduction in starch digestibility (Figure 2), EGIs and increase in RS (Table 4) of porridges were achieved with high tannin bran extract, followed by extracts from the bran of black with tannin and black sorghums.

**Table 4:** Estimated Glycemic Index (EGI) and Resistant Starch (RS) Contents of High Amylose Corn Starch Porridge Cooked with Sorghum Bran Extracts.

Porridges	Estimated Glycemic Index (EGI)	Resistant Starch (% dry weight basis)
High amylose corn starch	70.0± 3.0 ^a,1^	47.0± 2.0 ^c^
High amylose corn starch + white bran extract	70.0± 3.0 ^a^	46.0± 1.0 ^c^
High amylose corn starch + high tannin bran extract	48.0± 5.0 ^c^	58.0± 2.0 ^a^
High amylose corn starch + black bran extract	60.0± 3.0 ^b^	51.0± 4.0 ^b^
High amylose corn starch + black with tannin bran extract	60.0± 2.0 ^b^	53.0± 4.0 ^b^

^1^ The data represent the means ± SD of three determinations. Values with the same letter in one column are not significantly different (*p* = 0.05) from each other. Values are reported on a dry basis.

### 2.3. The Effects of 70% Aqueous Acetone Extracts from Sorghum Brans on Starch Digestibility of Whole Sorghum Flour Porridges

**Figure 3:**
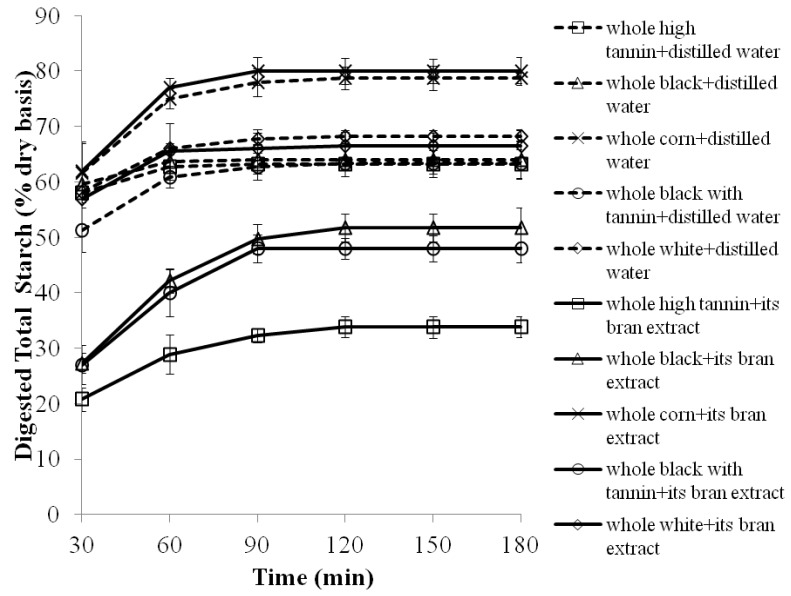
Starch digestibility of whole sorghum flour porridges made with sorghum bran extracts as a function of time (porridges used the equivalent of 50 mg of starch on a dry weight basis). Values are means ± standard error of *n* = 3 samples per treatment. Dash lines are controls, which are whole grain flours cooked with distilled water.

Ground whole sorghums were cooked with distilled water and 70% aqueous acetone extracts from the sorghum brans to determine if the additional phenolic content would contribute to additional reductions in starch digestibility. Whole sorghum porridges cooked with distilled water had significantly (*p* < 0.05) lower starch digestibility than whole white corn flour porridges. Phenolic-rich whole specialty sorghum porridges cooked with distilled water had lower (*p* < 0.05) starch digestibility after 180 min of digestion than whole white sorghum porridges (Figure 3).

Sorghum bran extracts, with the exception of the white sorghum bran, significantly (*p* < 0.05) decreased starch digestibility, EGI and significantly (*p* < 0.05) increased RS contents of whole sorghum porridges compared to whole white corn flour porridge cooked with corn bran extract (Figure 3, Table 5). Compared to sorghum porridges cooked with distilled water, whole sorghum flours cooked with their corresponding bran extracts (except white sorghum) significantly (*p* < 0.05) decreased starch digestibility, EGI and significantly (*p* < 0.05) increased RS contents of the porridges (Figure 3, Table 5). Whole high tannin porridge cooked with its bran extract had the lowest EGI and the highest RS, followed by whole black with tannin and black sorghum porridges (Table 5).

**Table 5:** Estimated Glycemic Index (EGI) and Resistant Starch (RS) contents of whole sorghum porridges cooked with distilled water or their corresponding bran extracts.

Porridges	EGI	RS (% dry weight basis)
Whole white corn cooked with water	100.0 ± 3.3 ^a,1^	0.1 ± 0.0 ^e^
Whole white corn cooked with its bran extract	98.0 ± 2.1 ^a^	0.0 ± 0.0 ^e^
Whole white sorghum cooked with water	92.0 ± 4.3 ^b^	0.2 ± 0.0 ^e^
Whole white sorghum cooked with its bran extract	91.0 ± 3.0 ^b^	0.3 ± 0.0 ^e^
Whole high tannin sorghum cooked with water	85.0 ± 4.5 ^c^	2.2 ± 1.2 ^c^
Whole high tannin sorghum cooked with its bran extract	62.0 ± 4.1 ^e^	6.8 ± 0.3 ^a^
Whole black sorghum cooked with water	88.0 ± 5.0 ^c^	0.3 ± 0.0 ^e^
Whole black sorghum cooked with its bran extract	73.0 ± 4.0 ^d^	2.4 ± 0.5 ^c^
Whole black with tannin sorghum cooked with water	87.0 ± 5.0 ^c^	1.0 ±0.2 ^d^
Whole black with tannin sorghum cooked with its bran extract	72.0 ± 4.3 ^d^	5.3 ± 0.4 ^b^

^1^ The data represent the means ± SD of three determinations. Values with the same letter in one column are not significantly different (*p* = 0.05) from each other. Values are reported on a dry basis.

### 2.4. Discussion

Perturbations in glycemic control contribute to significant health problems for diabetics and pre-diabetic subjects [4,5,26]. Devising appropriate food modifications that slow glucose release into the blood could facilitate patient compliance. One approach is to reduce starch digestibility through the creation of higher levels of resistant starch in foods [5,6,7]. The purpose of this study was to determine if extracts of tannin- or anthocyanin-containing brans from sorghum bran would elevate RS content of porridges and reduce starch digestibility. Extracts were prepared from specialty sorghum brans because they provide a condensed source of total phenols and condensed tannins, which would be a relatively inexpensive source of these compounds [24].

The phenol- and tannin-containing sorghum bran extracts reduced starch digestibility, EGI and increased RS content of both corn starch and high amylose corn starch porridges. The reduced EGI and elevated RS contents in these porridges were similar to, or improved, relative to those found with legumes, whole grain pasta or cereals [26]. However, the extract from white sorghum bran did not reduce starch digestibility or EGI, and did not increase RS content, which may be due to the low level of phenols and lack of detectable tannins in this material.

These data indicate that the polyphenolic molecules within the sorghum bran extracts were capable of reducing digestibility to a similar extent when corn starch or a high amylose corn starch was used. The effect of phenolics on starch digestibility could be two-fold; first, because phenolic can bind to starch (which can be seen with pasting profile, and stated [12,19,25,27], resulting in a different cooking and cooling viscosity profile, secondly phenolic can inhibit enzymes [18,25,28], which decreases the enzyme activity. Considering previous work has demonstrated that the use of high amylose corn starch reduces postprandial plasma glucose *in vivo* [29], we anticipate that similar *in vivo* reductions in blood glucose following a meal containing foods made with the tannin- or anthocyanin-containing extracts would produce similar physiological benefits.

Similar to what has been shown previously [28,29,30,31,32], our results suggest that polyphenols in the sorghum bran extracts limit enzyme activity, and leads to decreased starch digestibility, EGI, and increased RS contents of the porridges. To determine if it were possible to further suppress starch digestibility in products made with whole grain sorghum flour containing these polyphenols, we cooked whole sorghum grain flours with their corresponding bran extracts. In sorghum endosperm, like other cereals, the starch granules and protein bodies are embedded in a continuous protein matrix in the peripheral and corneous areas. Sorghum kafirins can form resilient web or sheet-like structures due to formation of disulfide cross links within and possibly between, protein bodies during cooking [33,34,35]. This interferes with digestive enzyme accessibility to gelatinized starch. As mentioned before, tannins form less extractable polymers either with other food components in particular proteins and carbohydrates or between themselves [9,10,13]. Tannin interactions with food components are mostly non-covalent interactions, and may involve hydrogen bonding and hydrophobic interactions. Sorghum tannins have strong affinity for proteins high in proline content like the prolamins [35]. Beta *et al.* [19] observed the reduced extractability as reduced measurable levels by methods such as the vanillin-HCl assay. This study showed that whole sorghum porridges had significantly (*p* < 0.05) lower starch digestibility than whole corn porridge. Whole tannin, black, and black with tannin sorghum porridges cooked with distilled water had significantly (*p* < 0.05) lower starch digestibility than whole white sorghum porridges, again demonstrating the dependence upon tannins and anthocyanins in these grains to reduce the rate of glucose release from starch in the grain.

Some assumptions are that during cooking condensed tannins are either destroyed or structurally modified. Dlamini [12] and Porter [36] reported that under heat and acidic conditions tannins depolymerize to oligomers and monomers, the basic phenolic structure remaining stable. Condensed tannins undergo polymerization at alkaline pH to form higher molecular weight polymers that are highly cross-linked and insoluble [12,19,36]. In this study, during pepsin digestion, de-polymerization of tannins may have occurred because of the one-hour protein digestion in a low pH pepsin solution (pH 1.5), which may have occurred in concert with tannins being freed from tannin-protein complexes [37]. Moreover, release of entrapped tannins from a starch gel could be promoted by alpha amylase digestion at a higher pH (pH 6.9). It is theoretically possible that during porridge digestion, tannin-protein and tannin-starch complexes were destabilized, which permitted polymerization among the phenolic molecules leading to enhanced enzyme inhibition.

Specialty sorghum bran extracts significantly (*p* < 0.05) decreased starch digestibility of whole sorghum porridges (*p* < 0.05). One hypothesis could be that the high amounts of phenols in aqueous medium would increase alpha amylase inhibition and promote phenolic compounds interactions with soluble gelatinized starch and denatured protein during and after cooking. Through these mechanisms the bran extracts may have further decreased starch digestibility, EGI, and increased RS contents in whole sorghum porridges.

## 3. Experimental

### 3.1. Sorghum Varieties

All sorghum varieties were collected at maturity (at 120 days, collected in August), air-dried, manually cleaned, glumes removed, and the cleaned grain was stored at −20 °C. Tannin sorghum varieties were high-tannin sorghum (CSC3xR28, College Station 2003) and black with tannin sorghum (Black PI Tall College Station TX, 2005). Non-tannin sorghum varieties were white food-type sorghum (ATx631*RTx436 College Station TX, 2003) and high anthocyanin black sorghum (TX430 black, College Station TX, USA, 2001). Homogenous 10 kg batches from each sorghum variety were prepared from 50 kg of mother batches to use in the analyses described below.

### 3.2. Control Samples and High Amylose Corn Starch

A whole white corn (Cargill Inc, Minneapolis, MN, USA) and a commercial corn starch (Argo) were used as controls. High amylose corn starch (Hi-maize 260, enzyme resistant) was obtained from National Starch & Chemical (Bridgewater, NY, USA). Whole white corn was milled to pass through a 1 mm screen using a UDY cyclone mill (Model 3010-030, UDY Corporation, Fort Collins, CO, USA). The samples were stored at −20 °C until used for analysis.

### 3.3. Preparations of Whole Sorghum Flours and Brans

Whole sorghum grains and whole white corn were milled to pass through a 1 mm screen using a UDY cyclone mill (Model 3010-030). Brans of sorghum varieties were obtained by decorticating 4-kg batches in a PRL mini-dehuller (Nutana Machine Co., Saskatoon, SK, Canada). The bran was then separated with a KICE grain cleaner (Model 6DT4-1, KICE Industries Inc., Wichita, KS, USA). The bran (approximately 10% of original grain weight) was further milled to pass through a 1 mm screen using a UDY cyclone mill (Model 3010-030) and stored at −20 °C.

### 3.4. Preparation of Extracts from Sorghum Brans

The milled brans (3 g) were extracted for 3 h using 30 mL of 70% aqueous acetone with constant shaking at low speed in an Eberbach shaker (Eberbach Corp., Ann Arbor, MI, USA) in quadruplicate. The extracts were centrifuged at 769 × *g* for 20 min and supernatants were collected. Acetone (reagent grade) was removed under vacuum at 30 °C, and total of 27 mL aqueous extracts were collected from each bran to cook the porridges. These aqueous bran extracts were added to corn starch, high amylose corn starch, and whole sorghum flours to make porridges. Extracts were kept at −20 °C in the freezer until the next day, and were brought to room temperature to make porridges. Tannin and phenol determinations were conducted on bran extracts and bran residues after extraction to obtain % extract recovery, which was calculated as below:



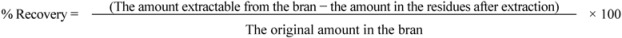



### 3.5. Tannin and Phenol Determination

A modified vanillin-HCl method [38] was used to determine tannin contents of whole sorghum grains and their brans. The milled samples were extracted at 30 °C for 20 min. The mixture was centrifuged for 10 min at 3,000 × *g*. A 1 mL volume of the supernatant was mixed with 5 mL vanillin reagent (4-hydroxy-3-methoxybenzaldehyde). The vanillin reagent (reagent grade) contained 4% concentrated HCl and 0.5% vanillin in methanol. The reaction was carried out in a 30 °C water bath for 20 min, and absorbance read at 500 nm after 20 min. Blank determinations were done to compensate for the color of the samples, by replacing the vanillin reagent with 4% HCl in methanol. The standard used was catechin (Sigma-Aldrich Inc., St Louis, MO, USA); tannin content was expressed as mg catechin equivalents per g (mg CE/g).

The Folin Ciocalteu method of Kaluza *et al.* [38], as modified by Dykes *et al.* [37], was used to determine total phenols of sorghum varieties. The milled samples were extracted for 2 h at low speed in an Eberbach shaker. One aliquot of the supernatant (0.1 mL) was diluted with water (1.1 mL) and was then reacted with Folin reagent (reagent grade, 0.4 mL) and 0.5 M ethanolamine (0.9 mL). The reaction was allowed to stand for 20 min at room temperature, and the absorbance was read at 600 nm. Gallic acid was used as a standard and the values are reported in gallic acid equivalents (mg/g dry basis).

The pH differential method of Fuleki and Francis [39] with modifications from Awika *et al.* [40] was used to estimate crude anthocyanin content of sorghum varieties and brans. One of two extract aliquots (0.2 mL) was diluted with acid (2.8 mL, pH 1.0, 125 mL of 0.2 N KCl, and 385 mL of 0.2 N HCl) and the other with pH 4.5 buffer (400 mL of 1 N sodium acetate, 240 mL of 1 N HCl, and 360 mL of distilled water). The absorbance was measured by scanning with a Cary 300 Bio UV-vis spectrophotometer (Varian Co., Walnut Creek, CA, USA) from 300 to 700 nm.

### 3.6. Preparation of Porridges

Corn starch (2.8 g, dry basis), high amylose corn starch (2.8 g, dry basis), and ground whole sorghum (3.5–4.5 g, dry basis, total starch equivalent) were cooked with extracts of bran from white, tannin, black and black with tannin sorghum varieties. Corn starch and high amylose corn starch cooked with only distilled water served as controls. Flours of whole sorghum and white corn cooked with distilled water were used as controls for whole flour porridges.

The samples above were cooked in 12 mL of the aqueous suspension of bran extracts in a Rapid Visco Analyser (RVA, Model RVA-4D, Newport Scientific, Narrabeen, Australia) interfaced with a personal computer equipped with Thermocline software. A programmed heating and cooling cycle was used, in which the mixture was held at 50 °C for 1 min, heated to 95 °C in 7.5 min at the rate of 6 °C/min, held at 95 °C for 5 min before cooling to 50 °C in 7.5 min and holding at 50 °C for 1 min. The porridges were cooled to 50 °C for 5 min, and left at room temperature for 10 min. An additional 13.1 mL of the aqueous suspension of bran extracts (a total of 25.1 mL aqueous extract together with first cooking) was added to the cooked porridge for the second cooking using the described cooking profile above. The porridges were cooled to 50 °C for 5 min, and left at room temperature for 10 min before further analysis. Preliminary studies conducted showed that double cooking of corn starch significantly (*p* < 0.05) decreased starch digestibility.

### 3.7. Total Starch, *in Vitro* Starch Digestibility, and Resistant Starch

Potentially available starch content was assessed by following the multienzymatic protocol of McCleary *et al*. [41]. The procedure and model established by Goñi *et al.* [42] was used to measure the *in vitro* starch hydrolysis. Triplicate samples of 50 mg starch (dry basis) equivalent wet porridges (as ready to eat) were homogenized in 10 mL of a HCl-KCl mixture (pH = 1.5) for 1 min using a Polytron homogenizer (Kinematica GmbH, Lucerna, Switzerland) with controlled speed (level 4). The samples were incubated at 40 °C for 60 min in a shaking water bath with 0.2 mL of a solution containing 1 mg of pepsin from porcine gastric mucosa (P-7000, Sigma-Aldrich Inc.). Fifteen mL of Tris-Maleate buffer (pH 6.9) was added to adjust pH. Then another 5 mL of Tris-Maleate buffer containing 2.6 UI of α-amylase from porcine pancreas (A-3176, Sigma-Aldrich Inc.) was added. The flasks were placed in a water bath at 37 °C with agitation. Aliquots (0.1 mL) were taken every 30 min from 0 to 3 h. α-Amylase was inactivated by immediately placing the tubes in a boiling water bath for 10 min with vigorous shaking every 30 s. Then, 1 mL of 0.4 M sodium-acetate buffer pH = 4.75 and 30 μL of amyloglucosidase from *Aspergillus niger* (A-1602, Sigma-Aldrich Inc.) were added. The samples were incubated at 60 °C for 45 min to hydrolyze the starch into glucose. Finally, the glucose concentration was measured using a glucose oxidase-peroxidase kit (Megazyme Int, Wicklow, Ireland). The experiment was repeated two times for each sample.

The rate of starch digestion was expressed as a percentage of total starch hydrolyzed at different times (30, 60, 90, 120, 150, and 180 min). The digestion curves were adjusted to the following non-linear equation established by Goñi *et al.* [42] to describe the kinetics of starch hydrolysis:



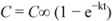



where *C* is the percentage of starch hydrolyzed at time *t* (min); *C*∞ is the equilibrium percentage of starch hydrolyzed after 180 min; and k is the kinetic constant. The variables *C*∞ and k were estimated for each sample using SPSS for Windows 11.5.

Hydrolysis Index and Estimated Glycemic Index (EGI):

From the digestion curves obtained during starch hydrolysis, the area under the hydrolysis curve (AUC) was calculated for each sample using the equation:



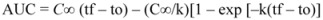



where tf is the final time (180 min) and to is the initial time (0 min); *C* is the percentage of starch hydrolyzed at time *t* (min); *C*∞ is the equilibrium percentage of starch hydrolyzed after 180 min; and k is the kinetic constant. The hydrolysis index (HI) was obtained by dividing the area under the hydrolysis curve of each sample by the corresponding area of a reference sample (fresh white bread, GI = 100) [42]. Finally, the estimated glycemic index (EGI) was predicted with the formula:



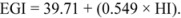



RS content in the food was measured using the method described by Goñi *et al.* [43]. In brief, the main steps of the procedure include removal of protein from samples with pepsin (Art.7190, Merck, Darmstadt, Germany) followed by α-amylase (A-3176, Sigma-Aldrich Inc.) incubation for 16 h to hydrolyze digestible starch. The hydrolysate was centrifuged for 10 min at 1,500 × *g* and the supernatant discarded. The residue is treated with 2M KOH for 1 h to solubilize resistant starch and the sample is then incubated with amyloglucosidase before glucose content is determined using a glucose oxidase assay (GOPOD reagent, Megazyme International). RS was calculated as glucose (mg) × 0.9 on dry basis.

### 3.8. Statistical Analyses

The data were analyzed using one way analysis of variance (ANOVA) using SPSS v 11.5 software (SPSS Inc., Chicago, IL, USA). Differences among treatments were detected using a Duncan’s test with a confidence level of 95% (α = 0.05). For physical and chemical characteristic of sorghum varieties, significance of differences within varieties and mean values of the cultivars, analysis of variance (ANOVA) and least significant differences (LSD, *p* = 0.05) were computed.

## 4. Conclusions

Overall, the phenol-containing extracts of specialty sorghum varieties used in this study significantly (*p* < 0.05) affected the rate and extent of starch digestibility of porridges. Significantly (*p* < 0.05) lower EGI and higher RS values were observed in porridges made with bran extracts containing high levels of phenolic compounds. These results demonstrate that the sorghum phenols, present mainly in the bran, have potential applications in food and pharmaceutical industries to decrease health problems related to type 2 diabetes and to reduce weight because of their ability to reduce starch hydrolysis.
